# The Characterization of the Mitochondrial Genome of *Fulgoraria rupestris* and Phylogenetic Considerations within the Neogastropoda

**DOI:** 10.3390/genes15081076

**Published:** 2024-08-14

**Authors:** Jiale Ma, Xiangli Dong, Kaida Xu, Jiaying Zeng, Zhongming Wang, Jiji Li

**Affiliations:** 1 Marine and Fisheries Institute of Zhejiang Ocean University, Zhoushan 316022, China; majiale@zjou.edu.cn (J.M.); 15924026180@163.com (J.Z.); wangzhongming@zjou.edu.cn (Z.W.); 2 National Engineering Research Center for Marine Aquaculture, Zhejiang Ocean University, Zhoushan 316022, China; dongxiangli@zjou.edu.cn; 3 Zhejiang Key Laboratory of Sustainable Utilization of Technology Research for Fisheries Resources, Scientific Observing and Experimental Station of Fishery Resources for Key Fishing Grounds, Ministry of Agriculture and Rural Affairs Zhejiang Marine Fisheries Research Institute, Zhoushan 316021, China

**Keywords:** mitochondrial genome, *Fulgoraria rupestris*, Neogastropoda, phylogenetic analysis

## Abstract

*Fulgoraria rupestris* is a predatory marine gastropod belonging to Neogastropoda and possessing considerable taxonomic significance. However, research on this species remains limited. We acquired the complete mitochondrial genome of *F. rupestris* through second-generation sequencing and conducted an analysis of its genome structural features. The mitochondrial genome of *F. rupestris* spans a total length of 16,223 bp and encompasses 37 genes (13 protein-coding genes (PCGs), 22 transfer RNAs, and 2 ribosomal RNAs). Notably, most tRNAs exhibit the typical cloverleaf structure, but there is an absence of the Dihydrouridine (DHU) arm in the *trnS1* and *trnS2* genes. The A + T content is 68.67%, indicating a pronounced AT bias. Additionally, we conducted a selection pressure analysis on the mitochondrial genomes of four species within Volutidae, revealing that all PCGs are subjected to purifying selection. In comparison to other species within Neogastropoda, *F. rupestris* shares an identical gene arrangement. Additionally, based on mitochondrial genome sequences of the 13 PCGs from 50 species within Neogastropoda, we constructed a phylogenetic tree. The phylogenetic tree indicates *F. rupestris* forms a clade with species within the family Volutidae (*Cymbium olla*, *Neptuneopsis gilchristi*, and *Melo melo*). This study serves as a valuable reference for future research on *F. rupestris*, offering insights for the upcoming phylogenetic and taxonomic classification within Neogastropoda. Furthermore, the findings provide valuable information for the development of genetic resources in this context.

## 1. Introduction

Neogastropoda, a taxonomically rich assemblage of marine predatory invertebrates, is systematically categorized into eight superfamilies: Buccinoidea, Conoidea, Mitroidea, Muricoidea, Olivoidea, Turbinelloidea, Volutoidea, and Pholidotomoidea (fossil only) [1,2]. Since the Cretaceous period, Neogastropoda has undergone prolific diversification, colonized nearly all of the world’s oceans, and asserted dominance within numerous shallow marine ecosystems [3,4]. An integral trait of Neogastropoda is its prominent predatory behavior, with most of its constituent species exhibiting carnivorous feeding strategies. The evolution of this behavior is attributed to significant morphological adaptations, which encompass the elongation of the proboscis, the relocation of the mouth to the anterior end of the head, and the development of highly specialized tentacles [5,6,7,8]. Given the extensive species diversity within Neogastropoda, its taxonomic classification has undergone significant modifications throughout its evolutionary history. The Neogastropoda are considered monophyletic in morphological classifications [9,10,11]. The phylogenetic relationships among Neogastropod families are quite unstable and highly controversial in molecular analyses [12,13]. The phylogenetic relationships among Neogastropod superfamilies remain unresolved [13].

The taxonomic classification of Volutoidea has also undergone numerous revisions. Wenz et al. (1938) [14] and Thiele et al. (1963) [15] relied on morphological and anatomical data to classify Volutoidea into Volutidae, Olividae, Mitridae, Turbinellidae, Harpidae, Marginellidae, and Cancellariidae families. Bouchet et al. (2017) [16], based on molecular data from Fedosov et al. (2015) [17], included only Volutidae and Cancellariidae in Volutoidea. Subsequently, Fedosov et al. (2019) [18], employing molecular phylogenetic analysis, provided support for the inclusion of four families (Volutidae, Cystiscidae, Marginellidae, and Marginellonidae) within the superfamily Volutoidea. According to the revision by the World Register of Marine Species (WoRMS: https://www.marinespecies.org/, accessed on 15 July 2024), Volutoidea is classified into Cancellariidae, Cystiscidae, Granulinidae, Marginellidae, Marginellonidae, and Volutidae.

Pilsbry et al. [19] initially classified Volutidae into 12 subfamilies [20]. In their recent taxonomy, Bouchet et al. [16] further delineated Volutidae into ten subfamilies, with two subfamilies recognized as extinct. According to WoRMS (accessed on 15 July 2024), Volutidae is divided into 76 genus-level taxonomic units. Moreover, due to convergent morphological characteristics and the plasticity influenced by environmental factors, the taxonomy and phylogeny of Volutidae have been perplexing [21]. When establishing classifications at levels such as families and genera, morphological features are susceptible to subjective interpretation by taxonomists.

Volutidae, a predatory marine gastropod within Neogastropoda [21], can trace its origins back to the late Early Cretaceous period [22,23,24]. Most species from Volutidae inhabit shallow soft-bottom substrates in tropical and temperate regions, with certain derived species also distributed in polar and deep-sea areas [25]. Many species within the Volutidae hold economic significance, being valuable components of luxury seafood and traditional medicines [26]. Recently, there has been an increase in research on Volutidae [27,28,29,30,31,32,33,34,35,36,37]. *Fulgoraria rupestris,* a species belonging to the Volutidae family (Neogastropoda: Volutoidea), exhibits a slightly hemispherical apex with an elliptical aperture. Its outer lip is distinguished by prominent thickness and distinctive wave-like features, often accompanied by brown markings on the outer wall [38]. However, literature surveys reveal a relatively limited amount of research on *F. rupestris*.

The rapid advancement of molecular biology techniques has made significant contributions to resolving complex issues in morphological classification [39,40,41,42]. Mitochondrial genes, known for their straightforward molecular structure, strict maternal inheritance, minimal recombination, and rapid evolutionary rate, have become valuable molecular markers. Particularly, complete mitochondrial genomes serve as excellent tools for studying phylogenetic relationships and taxonomic identification. Furthermore, compared to relying on individual mitochondrial genes, phylogenetic analysis based on the 13PCGs of mitochondrial genomes can enhance the resolution and statistical confidence of phylogenetic trees [43,44,45].

However, research on the complete mitochondrial genome of species within the Volutidae, including *F. rupestris*, is still limited. This study aims to elucidate the complete mitochondrial genome sequence of *F. rupestris*, analyze its basic nucleotide composition characteristics, explore interspecies evolutionary patterns, and construct a phylogenetic tree. We anticipate that the results of this study will markedly advance our understanding of the evolution and systematic classification within Neogastropoda, providing essential reference information for the development of genetic resources.

## 2. Materials and Method

### 2.1. Sample Preparation and DNA Extraction

One sample of *F. rupestris* was collected in November 2018 from the sea area of Zhoushan, Zhejiang, China (30°19′ N, 122°72′ E), using the bottom trawl technique [46]. Taxonomic specialists from the Museum of Marine Biology at Zhejiang Ocean University identified the specimen. Fresh tissues were dissected from the shell and after removing the digestive glands, the muscle tissues were stored in Ethanol absolute. Total DNA was extracted using the salt precipitation method [47].

### 2.2. Mitochondrial DNA Sequencing and Assembly

The mitochondrial genome is sequenced using the Illumina Novaseq^TM^ platform at Shanghai Yuanshen Biomedical Technology Co. Ltd. (Shanghai, China) Initially, the genomic DNA of the samples undergoes quality control. Upon passing quality control, the DNA is fragmented into 300–500 base-pair fragments through ultrasonication, followed by purification. Subsequently, sequencing libraries are constructed from the fragmented DNA. The steps encompass DNA end repair, A−tailing at the 3′ end, the ligation of sequencing adapters, gel electrophoresis for the recovery of target fragments, the PCR amplification of target fragments, and, ultimately, the construction of sequencing libraries. Prior to sequencing, the constructed library undergoes quality control. Once it passes the quality check, sequencing is performed using the Illumina Novaseq^TM^ platform. After obtaining raw data, filtering is carried out to exclude sequencing adapter sequences, low-quality reads, sequences with high N rates, and short-length sequences. This process results in high-quality sequencing data [48]. The preliminary assembly results are obtained using GetOrganelle (https://github.com/Kinggerm/GetOrganelle/, accessed on 1 October 2023) and the best assembly results are achieved through multiple correction iterations. The assembly of the mitochondrial genes of *F. rupestris* is validated by BLAST against the *cox1* barcode sequences in GenBank (https://www.ncbi.nlm.nih.gov/, accessed on 10 October 2023).

### 2.3. Genome Annotation and Bioinformatics Analysis

Genome annotation was conducted using the online tool MITOS (http://mitos2.bioinf.unileipzig.de/index.py, accessed on 6 January 2024) [49]. The invertebrate mitochondrial genetic code was selected and the usage of start and stop codons was compared with those of closely related relatives [20,26]. Following correction with Sequin, the complete mitochondrial genome data were uploaded to the NCBI database (https://www.ncbi.nlm.nih.gov/, accessed on 6 January 2024) to obtain the GenBank accession number. The circular mitochondrial genome map was generated using the online platform Proksee (https://proksee.ca/, accessed on 6 January 2024). DAMBE 7.0 [50] was employed to calculate the content of ATCG bases in the mitochondrial genome, as well as the content of the 13 protein-coding genes (PCGs). The skew values were computed using the formulas AT skew = (A − T)/(A + T) and GC skew = (G − C)/(G + C) [51]. MEGA X was used to calculate the frequency of amino acid usage and the relative synonymous codon usage (RSCU) in PCGs, while Ka/Ks (non-synonymous to synonymous substitutions) ratios were computed using DnaSP6.0 [52,53].

### 2.4. Phylogenetic Analysis

We constructed a phylogenetic tree based on the mitochondrial genome sequences of 50 species within Neogastropoda, including the newly sequenced *F. rupestris* and members of 5 superfamilies (Volutoidea, Buccinoidea, Conoidea, Muricoidea, and Olivoidea) downloaded from the NCBI database (Table 1). *Anodonta euscaphys* and *Anodonta arcaeformis* were included as outgroups with GenBank accession numbers KP187851 and KF667530, respectively. The phylogenetic analysis was conducted using the Bayesian inference (BI) method with MrBayes 3.2.7a and the maximum likelihood (ML) method with IQ−tree 2.1.3 [54,55]. Firstly, the 13 protein-coding gene sequences from 50 species were combined into a FASTA file and aligned by codon using the ClustalW algorithm in MEGA X software [52]. The aligned sequences were then trimmed, and the processed data were imported into the IQ-TREE program for analysis [55]. A chi-square test was performed, followed by the utilization of ModelFinder to automatically compute and select the best substitution model (GTR + F + R7) for constructing the ML tree [56,57]. Bayesian analysis was performed using MrBayes v3.2 [54], in conjunction with PAUP v4.0, Modeltest v3.7, and MrModeltest v2.3 from the MrMTgui v1.0 software. The best substitution model (GTR + I + G) was selected based on the AIC information criterion [58,59]. The BI tree was constructed using the Markov Chain Monte Carlo (MCMC) sampling method, with sampling every 1000 generations. The analysis ran for a total of 8 million generations, with the initial 25% of sampled data discarded as burn-in. The resulting consensus tree was obtained, and Posterior Probabilities (PPs) were calculated. Finally, the phylogenetic tree was visualized and edited using FigTree v1.4.3 and Adobe Photoshop 2019.

## 3. Results

### 3.1. Mitochondrial Genome Structural Features

**Figure 1:**
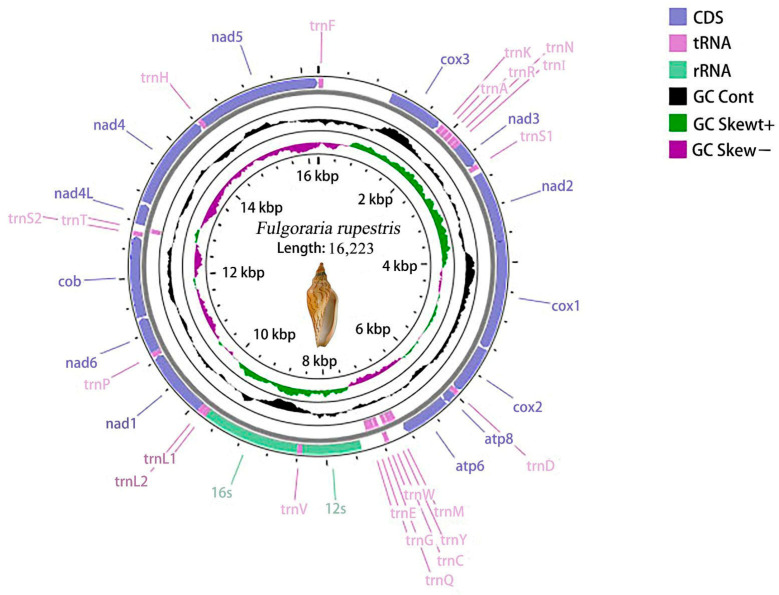
Complete mitogenome map of *F. rupestris*.

The mitochondrial genome of *F. rupestris* has been deposited in NCBI with GenBank accession number OR588873. The mitochondrial genome sequence of *F. rupestris* exhibits a classic circular configuration, with a length of 16,223 bp, encompassing 37 genes (Figure 1). These genes include 13 PCGs, 22 tRNAs, and 2 rRNAs (*12S rRNA* and *16S rRNA*). Among these genes, 29 genes are situated on the plus strand, while 8 genes are positioned on the minus strand (Figure 1, Table 2). The range of base pairs for the 13 PCGs is from 159 bp (*atp8*) to 1722 bp (*nad5*) (Table 2). In the mitochondrial genome of *F. rupestris*, the largest overlapping region is 23 bp between *nad2* and *cox1*, while the maximum intergenic nucleotide region is 122 base pairs between *trnE* and *12S rRNA*.

### 3.2. Analysis of rRNA and tRNA in the F. rupestris Mitochondrial Genome

**Figure 2:**
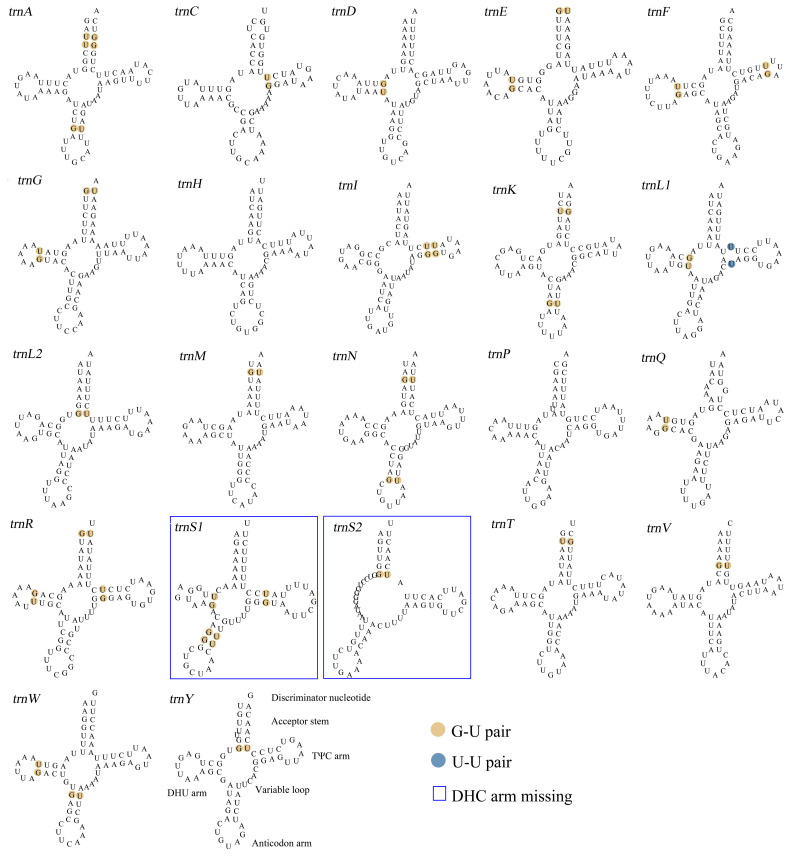
The secondary structure of *F. rupestris* mitochondrial tRNA.

The mitochondrial genome of *F. rupestris* encompasses two rRNA genes, namely *12S rRNA*, spanning 833 bp in length and *16S rRNA* and measuring 1,366 bp. *12S rRNA* is situated between *trnE* and *trnV*, while the *16S rRNA* gene is positioned between *trnV* and *trnL* (Table 2). The 22 tRNA genes collectively cover a sequence length of 1,490 bp, with individual lengths ranging from 65 to 70 bp. Notably, *trnY*, *trnC*, and *trnQ* each comprise 65 bp, while *trnF* and *trnP* each extend to 70 bp. Furthermore, *trnL* and *trnS* each possess two copies. Except for the absence of the DHU arm in *trnS1* and *trnS2*, the remaining 20 tRNA genes exhibit the typical cloverleaf secondary structure (Figure 2). Intriguingly, *trnL1* presents a U−U mismatch in the TΨC stem. Moreover, apart from *trnH*, *trnP*, and *trnS*, all other tRNA species display G−U mismatches. Specifically, *trnA*, *trnE*, *trnG*, *trnK*, *trnL2*, *trnM*, *trnN*, *trnR*, *trnS2*, *trnT*, *trnV*, and *trnY* exhibit G−U mismatches in the aminoacyl stem. Furthermore, *trnD*, *trnE*, *trnF*, *trnG*, *trnL1*, *trnQ*, *trnR*, and *trnW* display G−U mismatches in the DHC loop. Notably, *trnA*, *trnK*, *trnN*, *trnS1*, and *trnW* show G−U mismatches in the anticodon stem.

### 3.3. Nucleotide Composition and Base Skew Analysis

The nucleotide composition analysis of the mitochondrial genome in *F. rupestris* reveals distinct patterns. The respective percentages for each nucleotide are as follows: A at 30.55%, T at 38.12%, G at 16.01%, and C at 15.32% (Table 3). The cumulative A + T content stands at 68.67%, surpassing the G + C content of 31.33%. Notably, AT base pairs predominate, as evidenced by an AT-skew value of −0.11, indicating a subtle bias toward T, while the GC-skew value of 0.02 suggests a preference for G. Deeper insights emerge from the analysis of individual PCGs. The A content spans from 23.08% to 37.02%, T from 33.20% to 43.31%, G from 12.38% to 21.79%, and C from 11.94% to 17.57%. AT-skew values range from −0.26 to −0.05, while GC-skew values range from −0.13 to −0.20. Particularly noteworthy is the *nad2* gene, exhibiting a notably low C content (11.94%) and a higher G content (18.06%), resulting in an elevated G-base skew rate of 0.20.

### 3.4. Amino Acid Composition and Codon Usage

**Figure 3:**
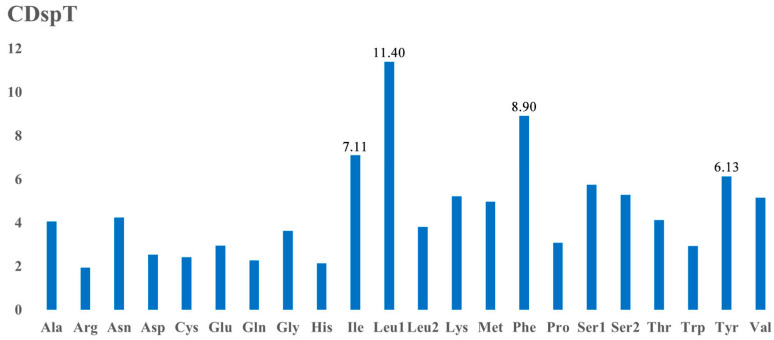
Amino acid composition in the mitochondrial genome of *F. rupestris*.

**Figure 4:**
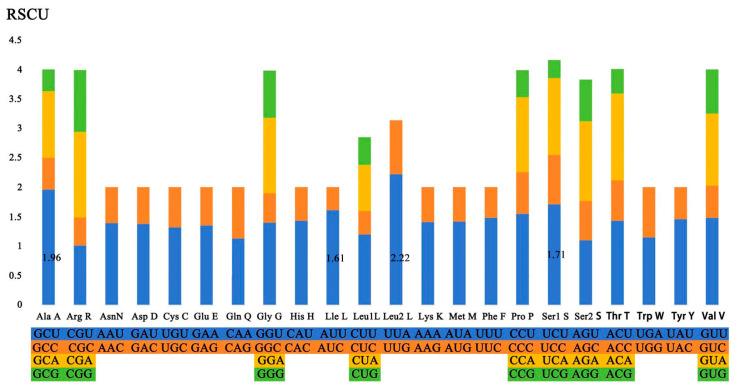
RSCU in the mitochondrial genome of *F. rupestris*.

The analysis of amino acid content reveals that Leu1, Phe, Ile, and Tyr are the four most prevalent amino acids, constituting 11.40%, 8.90%, 7.11%, and 6.13%, respectively (Figure 3). According to the RSCU values of the 13 PCGs, it was found that UUA (Leu2), GCU (Ala), UCU (Pro), and AUU (Ile) are the most frequently used codons, with UUA at 2.22%, GCU at 1.96%, UCU at 1.71%, and AUU at 1.61% (Figure 4). The analysis of start and stop codons for PCGs indicates that, apart from *nad2* and *nad5*, which commence with ATT, the rest of the genes initiate with ATG and terminate with TAA or TAG as stop codons.

### 3.5. Selection Pressure Analysis

**Figure 5:**
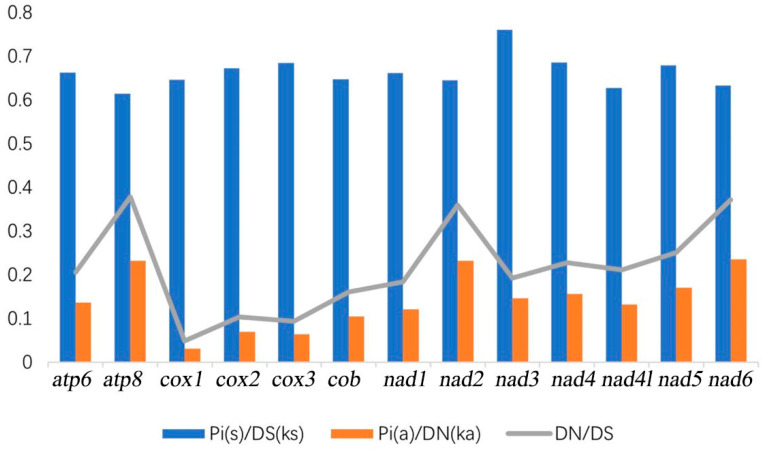
Analysis of the selection pressure of Volutidae; ka refers to non-synonymous substitution value, and ks refers to synonymous substitution value.

We selected mitochondrial genomes of four species from Volutidae to analyze selection pressure. The calculated Ka/Ks values for all 13 PCGs are below 1 (Figure 5). Notably, *atp8* exhibits the highest value at 0.38, while *cox1* has the lowest value at 0.05. The overall Ka/Ks ratio below 1 implies that mutations have predominantly led to synonymous substitutions, indicating a purifying selection impact on Volutidae species throughout their evolutionary history.

### 3.6. Gene Order

**Figure 6:**
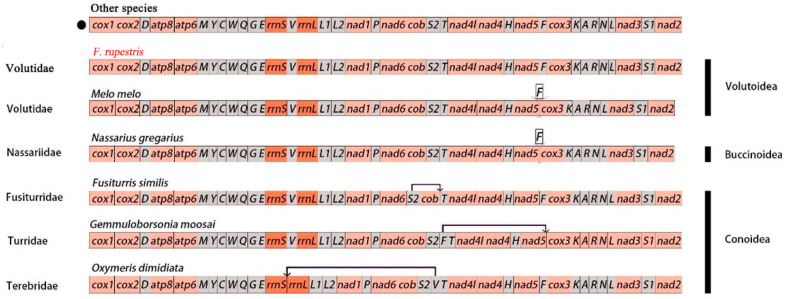
The black circles represent the mitochondrial gene sequences of the remaining 44 species.

Comparing the gene order of mitochondrial genomes of 50 species within Neogastropoda (Table 1), including *F. rupestris*, reveals that the gene order has changed in *M. melo*, *N. gregarious*, *F. similis*, *G. moosai*, and *O. dimidiata*, while the gene order is consistent among the remaining 44 species (Figure 6). *M. melo* and *N. gregarius* lack the *trnF* gene. *F. similis* underwent a gene inversion at *trnS2*−*cob*, resulting in the gene sequence becoming *cob*−*trnS2*.*G. moosai* presents a distinct order in the *trnF*−*trnT*−*nad4l*−*nad4*−*trnH*−*nad5* gene segment, differing from the *trnT*−*nad4l*−*nad4*−*trnH*−*nad5*−*trnF* order observed in other species. Notably, *trnF* has undergone transposition. The gene order of *O. dimidiata* shows a transposition of *trnV*, with the order rearranged to *rrnL*–*trnL1*–*trnL2*–*nad1*–*trnP–nad6*–*cob*–*trnS2*–*trnV*. Importantly, no mitochondrial genome rearrangements were observed in the 13 PCGs of other species.

### 3.7. Phylogenetic Relationships

**Figure 7:**
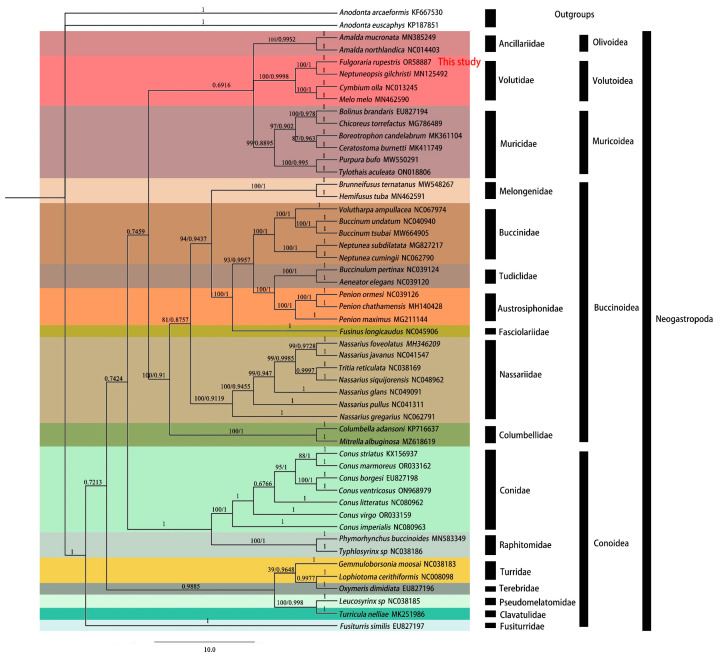
The phylogenetic tree, constructed based on the 13 PCGs of 50 Neogastropoda species, displays support values (BI, ML) for each node. (ML/BI; the range of ML support values is from 0 to 100, while the range of BI support values is from 0 to 1.)

We performed a phylogenetic analysis on the 13 PCG sequences extracted from 50 species, encompassing 5 superfamilies (i.e., Volutoidea, Buccinoidea, Conoidea, Muricoidea, and Olivoidea) within the Neogastropoda. Based on two methods (ML and BI), nearly identical topologies were obtained. *A. euscaphys* and *A. arcaeformis* were chosen as outgroups in constructing the phylogenetic tree (Figure 7).

The consolidation of Neogastropoda as a monophyletic group received substantial support from robust statistical values. In this analysis, *F. rupestris* formed a highly supported clade alongside *C. olla*, *N. gilchristi*, and *M. melo*. Buccinoidea, Muricoidea, Volutoidea, and Olivoidea clustered together to form a branch (bootstrap probability of 0.7459). Within this branch, the bootstrap support value for the relationship among Muricoidea, Volutoidea, and Olivoidea is 0.6916.

Based on the extensive mitochondrial genome data acquired in this study, the phylogenetic relationships within the primary lineage of Buccinoidea can be delineated as follows: ((Melongenidae + (((Buccinidae + (Tudiclidae + Austrosiphonidae)) + Fasciolariidae) + Nassariidae) + Columbellidae)). Within Conoidea, a tripartite division was observed, with Conidae and Raphitomidae forming a distinct cluster. Concurrently, Turridae, Terebridae, Pseudomelatomidae, Clavatulidae, and Fusiturridae constituted another discernible group. Fusiturridae occupied a basal position as an independent branch. However, the monophyly of these branches within Conoidea did not receive robust support.

## 4. Discussion

### 4.1. Basic Features of the Mitogenome of F. rupestris

*Fulgoraria rupestris*, like most gastropods, possesses a mitochondrial genome consisting of 37 genes [60,61,62]. The sequence lengths of the other three species (*M. melo*, *N. gregarious*, and *C. olla*) range from 15,312 to 15,721 base pairs. In the mitochondrial genome of *F. rupestris*, there is a *D-loop* region spanning 975 base pairs in length between *trnF* and *cox3*, which results in a total mitochondrial genome length of 16,223 bp in *F. rupestris*. The genome composition exhibits a pronounced AT bias, consistent with findings reported in previous studies [63,64,65].

Mitochondrial genes, with their high conservation, limited recombination, and maternal inheritance, are utilized to elucidate the evolutionary relationships among various animal taxa [66,67]. In contrast to vertebrate mitochondrial genomes, those found in mollusks demonstrate notable heterogeneity in both length and structure. This variability is attributed to disparities in gene loss or duplication, as well as variations in the position and strand specificity of tRNA, protein-coding, and rRNA genes [68]. In this study, among the four known species sequences within Volutidae, three exhibit a consistent gene order, while *M. melo* lacks *trnF*. This deletion in the non−coding region may be attributed to slippage events occurring in regions with high A/T or AT/TA repeats.

### 4.2. Phylogenetic Analysis

The classification of Neogastropoda remains contentious. This study is consistent with the taxonomic research by Bouchet et al. [16], emphasizing the monophyly of Buccinoidea, Muricoidea, Volutoidea, and Olivoidea [69]. Olivoidea, Volutoidea, and Muricoidea are closely related. This is consistent with the findings of Lemarcis et al. [70]. This study follows the classification by Harasewych et al. [25] and Fedosov et al. [16]. In the phylogenetic tree, Volutidae forms a clade, with *F. rupestris* being most closely related to *N. gilchristiy*. *F. rupestris* and *N. gilchristiy* exhibit greater morphological similarities.

Muricoidea, the second largest family in Neogastropoda, has not consistently exhibited monophyly in prior morphological and molecular studies [1,13,71,72]. Barco et al. [73] conducted a study based on partial sequences of three mitochondrial genes (*12S rRNA*, *16S rRNA*, and *COI*) and one nuclear gene (*28S rRNA*). Their analysis, employing Bayesian inference and maximum likelihood methods, supported the monophyly of Muricoidea [73]. Previous morphological and molecular studies have not adequately validated the monophyly of Buccinoidea [1,13,71]. Kantor et al. [74] found that Buccinoidea is monophyletic in Bayesian analysis but lacks support in ML analysis. Additionally, Galindo et al. confirmed Buccinoidea’s monophyly, yet further investigation is required to validate this assertion [75]. This study restored the monophyly of Buccinoidea, similar to the results of Oliverio et al., who used mitochondrial sequences (*16S rRNA*, *12S rRNA*, and *COI*) for their analysis [72]. Consistent with the study by Kantor et al., Austrosiphonidae and Tudiclidae are sister groups [74]. Cominellina was originally a subfamily within Buccinidae. Kantor et al. found that Cominellina has no affinity with Buccinidae and is not included in any larger supported clusters within the core Buccinoidea [74]. Therefore, it has been elevated to the rank of family and named Cominellidae. Our study has demonstrated this. In molecular phylogenetic investigations conducted by Puillandre et al. [76] and Yang et al. [77], Conoidea was identified as a monophyletic group, contrasting with the findings of our study. In our study, Conoidea is divided into three branches. This observation is consistent with the findings of Cunha et al. [13] and Zou et al. [21]. The instability or conflicting branches observed within Conoidea may be attributed to the limited sampling of taxonomic units.

Although the number of species increased to 50 in this study, the internal phylogenetic relationship of Neogastropoda is still uncertain. Further comprehensive mitochondrial DNA sequencing of additional gastropod lineages is necessary to effectively address this issue. However, the rapid diversification at the origin of Neogastropoda and the complex evolutionary patterns of genes associated with morphological differentiation may also complicate phylogenetic inference [78]. Therefore, additional nuclear sequence data need to be incorporated into phylogenetic analyses [13]. Further research is required to elucidate the phylogenetic positions of each superfamily within Neogastropoda.

## 5. Conclusions

This study conducted a comprehensive analysis of the complete mitochondrial genome of *F. rupestris* using molecular biology methods. The analysis encompassed the examination of mitochondrial genome content, organization, codon usage, gene arrangement, phylogenetic relationships, and positive selection. Based on the complete mitochondrial genome sequences, we constructed a phylogenetic tree of Neogastropoda. The study elucidated the phylogenetic relationships of *F. rupestris* with other species in the Volutidae family, including *C*. *olla*, *N*. *gilchristi*, and *M*. *melo*. This further confirms that *F. rupestris* belongs to the Volutidae family and contributes to enriching the genetic database. These findings establish a framework for species identification and evolutionary analysis within the Volutidae family and offer theoretical support for the future sustainable development and molecular breeding of *F. rupestris*.

## Figures and Tables

**Table 1:** List of species analyzed in this study and their GenBank accession numbers, with the newly sequenced *Fulgoraria rupestris* species marked with an asterisk (*).

Superfamily	Family	Species	Size (bp)	Accession No.
Olivoidea	Ancillariidae	*Amalda mucronata*	15,353	MN385249
		*Amalda northlandica*	15,354	NC014403
Volutoidea	Volutidae	*Fulgoraria rupestris* *	16,223	OR588873
		*Cymbium olla*	15,375	NC013245
		*Neptuneopsis gilchristi*	15,312	MN125492
		*Melo melo*	15,721	MN462590
Buccinoidea	Melongenidae	*Brunneifusus ternatanus*	16,254	MW548267
		*Hemifusus tuba*	15,483	MN462591
	Buccinidae	*Volutharpa ampullacea*	16,177	NC067974
		*Buccinum undatum*	15,265	NC040940
		*Buccinulum pertinax*	15,247	NC039124
		*Penion ormesi*	15,234	NC039126
		*Aeneator elegans*	15,254	NC039120
		*Buccinum tsubai*	15,262	MW664905
		*Penion chathamensis*	15,227	MH140428
		*Neptunea subdilatata*	15,393	MG827217
		*Penion maximus*	15,249	MG211144
		*Neptunea cumingii*	15,254	NC062790
	Nassariidae	*Nassarius foveolatus*	15,343	MH346209
		*Nassarius javanus*	15,325	NC041547
		*Nassarius pullus*	15,278	NC041311
		*Tritia reticulata*	15,337	NC038169
		*Nassarius siquijorensis*	15,337	NC048962
		*Nassarius glans*	15,296	NC049091
		*Nassarius gregarius*	15,171	NC062791
	Fasciolariidae	*Fusinus longicaudus*	16,319	NC045906
	Columbellidae	*Columbella adansoni*	16,272	KP716637
		*Mitrella albuginosa*	16,244	MZ618619
Conoidea	Conidae	*Conus striatus*	15,738	KX156937
		*Conus borgesi*	15,536	EU827198
		*Conus imperialis*	15,505	NC080963
		*Conus litteratus*	15,569	NC080962
		*Conus marmoreus*	15,579	OR033162
		*Conus virgo*	15,594	OR033159
		*Conus ventricosus*	16,307	ON968979
	Fusiturridae	*Fusiturris similis*	15,595	EU827197
	Turridae	*Gemmuloborsonia moosai*	15,541	NC038183
		*Lophiotoma cerithiformis*	15,380	NC008098
	Pseudomelatomidae	*Leucosyrinx* sp.	15,358	NC038185
	Terebridae	*Oxymeris dimidiata*	16,513	EU827196
	Raphitomidae	*Phymorhynchus buccinoides*	15,764	MN583349
		*Typhlosyrinx* sp.	15,804	NC038186
	Clavatulidae	*Turricula nelliae*	16,453	MK251986
Muricoidea	Muricidae	*Bolinus brandaris*	15,380	EU827194
		*Boreotrophon candelabrum*	15,265	MK361104
		*Ceratostoma burnetti*	15,334	MK411749
		*Chicoreus torrefactus*	15,359	MG786489
		*Purpura bufo*	15,239	MW550291
		*Tylothais aculeata*	17,024	ON018806
Unionoidea	Unionidae	*Anodonta euscaphys*	15,741	KP187851
		*Anodonta arcaeformis*	15,672	KF667530

**Table 2:** Organization of the mitogenome of *F. rupestris*.

Gene	Position(bp)		Direction	Length (bp)	Intergenic Nucleotides (bp)	Start/Stop Codons	Anticodon
	From	To					
*trnF*	1	70	+	70	0		GAA
*D−loop*	71	1,046	+	976	0		
*cox3*	1,047	1,826	+	780	18	ATG/TAG	
*trnK*	1,845	1,910	+	66	5		TTT
*trnA*	1,916	1,984	+	69	7		TGC
*trnR*	1,992	2,060	+	69	14		TCG
*trnN*	2,075	2,142	+	68	6		GTT
*trnI*	2,149	2,214	+	66	3		GAT
*nad3*	2,218	2,571	+	354	0	ATG/TAA	
*trnS1*	2,572	2,639	+	68	0		GCT
*nad2*	2,640	3,719	+	1,080	−23	ATT/TAA	
*cox1*	3,697	5,232	+	1,536	16	ATG/TAA	
*cox2*	5,249	5,935	+	687	−2	ATG/TAA	
*trnD*	5,934	6,002	+	69	1		GTC
*atp8*	6,004	6,162	+	159	6	ATG/TAA	
*atp6*	6,169	6,864	+	696	37	ATG/TAG	
*trnM*	6,902	6,967	−	66	2		CAT
*trnY*	6,970	7,032	−	65	13		GTA
*trnC*	7,046	7,110	−	65	0		GCA
*trnW*	7,111	7,178	−	68	−2		TCA
*trnQ*	7,177	7,241	−	65	10		TTG
*trnG*	7,252	7,318	−	67	0		TCC
*trnE*	7,319	7,386	−	68	122		TTC
*12SrnA*	7,509	8,341	+	833	−3		
*trnV*	8,339	8,407	+	69	4		TAC
*16SrnA*	8,412	9,777	+	1,366	−3		
*trnL1*	9,775	9,843	+	69	2		TAG
*trnL2*	9,846	9,914	+	69	1		TAA
*nad1*	9,916	10,857	+	942	4	ATG/TAG	
*trnP*	10,862	10,931	+	70	1		TGG
*nad6*	10,933	11,433	+	501	13	ATG/TAA	
*cob*	11,447	12,586	+	1,140	9	ATG/TAG	
*trnS2*	12,596	12,661	+	66	8		TGA
*trnT*	12,670	12,738	−	69	21		TGT
*nad4L*	12,760	13,056	+	297	−7	ATG/TAG	
*nad4*	13,050	14,423	+	1,338	3	ATG/TAA	
*trnH*	14,427	14,495	+	69	0		GTG
*nad5*	14,496	16,214	+	1,722	8	ATT/TAA	

**Table 3:** Base content in the mitogenome of *F. rupestris*.

*F. rupestris*	A (%)	T (%)	G (%)	C (%)	A + T (%)	G + C (%)	AT-Skew	GC-Skew
Mitogenome	30.55	38.12	16.01	15.32	68.67	31.33	−0.11	0.02
*cox1*	26.82	37.70	18.62	16.86	64.52	35.48	−0.17	0.05
*cox2*	30.71	35.37	17.76	16.16	66.08	33.92	−0.07	0.05
*atp8*	34.59	38.99	13.84	12.58	73.58	26.42	−0.06	0.05
*atp6*	26.72	42.39	14.08	16.81	69.11	30.89	−0.23	−0.09
*cox3*	23.08	38.97	21.79	16.15	62.05	37.95	−0.26	0.15
*nad3*	25.71	39.55	20.06	14.69	65.25	34.75	−0.21	0.15
*nad1*	28.24	39.49	15.61	16.67	67.73	32.27	−0.17	−0.03
*nad5*	29.55	39.44	13.44	17.57	68.99	31.01	−0.14	−0.13
*nad4*	29.62	40.61	13.03	16.74	70.23	29.77	−0.16	−0.12
*nad4l*	28.96	38.72	18.18	14.14	67.68	32.32	−0.14	0.13
*nad6*	28.74	43.31	12.38	15.57	72.06	27.94	−0.20	−0.11
*cob*	26.93	39.04	18.62	16.86	65.96	35.48	−0.18	0.05
*nad2*	28.24	41.76	18.06	11.94	70.00	30.00	−0.19	0.20
tRNAs	35.69	34.68	17.14	12.50	70.36	29.64	0.01	0.16
rRNAs	37.02	33.20	17.19	12.60	70.21	29.79	0.05	0.15
PCGs	27.84	39.37	16.59	16.21	67.21	32.80	−0.17	0.01

## Data Availability

*F. rupestris* mitogenome sequence data were deposited in GenBank with accession number OR588873.

## References

[1] Ponder W.F., Colgan D.J., Healy J., Nützel A., Simone L.R., Strong E.E. (2008). Caenogastropod phylogeny. Molluscan Phylogeny.

[2] Bernard F. (1890). Recherches sur les Organes Palléaux des Gastéropodes Prosobranches.

[3] Bouchet P., Lozouet P., Maestrati P., Heros V. (2002). Assessing the magnitude of species richness in tropical marine environments: Exceptionally high numbers of molluscs at a New Caledonia site. Biol. J. Linn. Soc..

[4] Ponder W.F. (1998). Brief introductions to higher groups of gastropods: Infraorder Neogastropoda. Mollusca: The Southern Synthesis.

[5] Ponder W., Lindberg D.R. (2008). Phylogeny and Evolution of the Mollusca.

[6] Kantor Y.I., Taylor J.D., Taylor J.D. (1996). Phylogeny and Relationships of Neogastropoda.

[7] Ponder W.F., Lindberg D.R. (1997). Towards a phylogeny of gastropod molluscs: An analysis using morphological characters. Zool. J. Linn. Soc..

[8] Strong E.E. (2003). Refining molluscan characters: Morphology, character coding and a phylogeny of the Caenogastropoda. Zool. J. Linn. Soc..

[9] Bouvier E. (1887). Système Nerveux, Morphologie Générale et Classification des Gastéropodes Prosobranches.

[10] Colgan D.J., Ponder W.F., Eggler P.E. (2000). Gastropod evolutionary rates and phylogenetic relationships assessed using partial 28S rDNA and histone H3 sequences. Zool. Scr..

[11] Colgan D.J., Ponder W.F., Beacham E., Macaranas J.M. (2003). Gastropod phylogeny based on six segments from four genes representing coding or non-coding and mitochondrial or nuclear DNA. Molluscan Res..

[12] Jiang X., Miao J., Li J., Ye Y. (2024). Characterization of Lophiotoma leucotropis Mitochondrial Genome of Family Turridae and Phylogenetic Considerations within the Neogastropoda. Animals.

[13] Cunha R.L., Grande C., Zardoya R. (2009). Neogastropod phylogenetic relationships based on entire mitochondrial genomes. BMC Evol. Biol..

[14] Schindewolf O.H. (1938). Handbuch der Paläozoologie. Nature.

[15] Thiele J. (1963). Handbuch der Systematischen Weichtierkunde.

[16] Bouchet P., Rocroi J., Hausdorf B., Kaim A., Kano Y., Nützel A., Parkhaev P., Schrödl M., Strong E.E. (2017). Revised classification, nomenclator and typification of gastropod and monoplacophoran families. Malacologia.

[17] Fedosov A., Puillandre N., Kantor Y., Bouchet P. (2015). Phylogeny and systematics of *Mitriform gastropods* (Mollusca: Gastropoda: Neogastropoda). Zool. J. Linn. Soc..

[18] Fedosov A.E., Caballer Gutierrez M., Buge B., Sorokin P.V., Puillandre N., Bouchet P. (2019). Mapping the missing branch on the neogastropod tree of life: Molecular phylogeny of *Marginelliform gastropods*. J. Molluscan Stud..

[19] Pilsbry H.A., Olsson A.A., Ithaca N.P.R.I. (1954). Systems of the Volutidae.

[20] Harasewych M.G., Sei M., Wirshing H.H., González V.L., Uribe J.E. (2019). The complete mitochondrial genome of *Neptuneopsis gilchristi* GB Sowerby III, 1898 (Neogastropoda: Volutidae: Calliotectinae). Nautilus.

[21] Zou S., Li Q., Kong L. (2011). Additional gene data and increased sampling give new insights into the phylogenetic relationships of Neogastropoda, within the caenogastropod phylogenetic framework. Mol. Phylogenet. Evol..

[22] Bandel K., Dockery Iii D.T. (2012). Protoconch characters of Late Cretaceous Latrogastropoda (Neogastropoda and Neomesogastropoda) as an aid in the reconstruction of the phylogeny of the Neogastropoda. Freib. Forschungshefte C.

[23] Modica M.V., Holford M. (2010). The Neogastropoda: Evolutionary innovations of predatory marine snails with remarkable pharmacological potential. Evolutionary Biology–Concepts, Molecular and Morphological Evolution: 13th Meeting 2009.

[24] Uribe J.E., Fedosov A.E., Murphy K.R., Sei M., Harasewych M.G. (2021). The complete mitochondrial genome of *Costapex baldwinae* (Gastropoda: Neogastropoda: Turbinelloidea: Costellariidae) from the Caribbean Deep-Sea. Mitochondrial DNA Part B.

[25] Harasewych M.G., Sei M., Uribe J.E. (2020). The complete mitochondrial genome of *Harpovoluta charcoti* (Gastropoda: Neogastropoda: Volutidae). Mitochondrial DNA Part B.

[26] Zhong S., Huang G., Liu Y., Huang L. (2019). The complete mitochondrial genome of marine gastropod *Melo melo* (neogastropoda: Volutoidea). Mitochondrial DNA Part B.

[27] Giménez J., Brey T., Mackensen A., Penchaszadeh P.E. (2004). Age, growth, and mortality of the prosobranch *Zidona dufresnei* (Donovan, 1823) in the Mar del Plata area, south-western Atlantic Ocean. Mar. Biol..

[28] Bigatti G., Penchaszadeh P.E. (2005). Imposex in *Odontocymbiola magellanica* (Caenogastropoda: Volutidae) in Patagonia. Comun. Soc. Malacol. Urug..

[29] Cledón M., Arntz W., Penchaszadeh P.E. (2005). Gonadal cycle in an *Adelomelon brasiliana* (Neogastropoda: Volutidae) population of Buenos Aires province, Argentina. Mar. Biol..

[30] Cledón M., Brey T., Penchaszadeh P.E., Arntz W. (2005). Individual growth and somatic production in *Adelomelon brasiliana* (Gastropoda; Volutidae) off Argentina. Mar. Biol..

[31] Gimenez J., Lasta M., Bigatti G., Penchaszadeh P.E. (2005). Exploitation of the volute snail *Zidona dufresnei* in Argentine waters, southwestern Atlantic Ocean. J. Shellfish Res..

[32] Cledón M., Theobald N., Gerwinski W., Penchaszadeh P. (2006). Imposex and organotin compounds in marine gastropods and sediments from the Mar del Plata coast, Argentina. J. Mar. Biol. Assoc. UK.

[33] Bigatti G., Carranza A. (2007). Phenotypic variability associated with the occurrence of imposex in *Odontocymbiola magellanica* from Golfo Nuevo, Patagonia. J. Mar. Biol. Assoc. UK.

[34] Bigatti G., Ciocco N.F. (2008). Volutid snails as an alternative resource for artisanal fisheries in northern patagonic gulfs: Availability and first suggestions for diving catches. J. Shellfish Res..

[35] Penchaszadeh P.E., Antelo C.S., Zabala S., Bigatti G. (2009). Reproduction and imposex in the edible snail *Adelomelon ancilla* from northern Patagonia, Argentina. Mar. Biol..

[36] Márquez F., González-José R., Bigatti G. (2011). Combined methods to detect pollution effects on shell shape and structure in Neogastropods. Ecol. Indic..

[37] Roche A., Maggioni M., Narvarte M. (2011). Predation on egg capsules of *Zidona dufresnei* (Volutidae): Ecological implications. Mar. Biol..

[38] Schumacher C.F. (1817). Essai d’un Nouveau Système des Habitations des vers Testacés: Avec XXII Planches.

[39] Manoylov K.M. (2014). Taxonomic identification of algae (morphological and molecular): Species concepts, methodologies, and their implications for ecological bioassessment. J. Phycol..

[40] Hajibabaei M., Singer G.A., Hebert P.D., Hickey D.A. (2007). DNA barcoding: How it complements taxonomy, molecular phylogenetics and population genetics. Trends Genet..

[41] Ryan U., Xiao L. (2013). Taxonomy and molecular taxonomy. Cryptosporidium: Parasite and Disease.

[42] Fontanilla I.K., Naggs F., Wade C.M. (2017). Molecular phylogeny of the achatinoidea (mollusca: Gastropoda). Mol. Phylogenet. Evol..

[43] Ingman M., Kaessmann H., Pääbo S., Gyllensten U. (2000). Mitochondrial genome variation and the origin of modern humans. Nature.

[44] Mueller R.L. (2006). Evolutionary rates, divergence dates, and the performance of mitochondrial genes in Bayesian phylogenetic analysis. Syst. Biol..

[45] Miao J., Feng J., Liu X., Yan C., Ye Y., Li J., Xu K., Guo B., Lü Z. (2022). Sequence comparison of the mitochondrial genomes of five brackish water species of the family Neritidae: Phylogenetic implications and divergence time estimation. Ecol. Evol..

[46] Patrice B. (2008). A new species of *Fulgoraria Schumacher*, 1817 (Gastropoda: Volutidae) from the bathyal Taiwanese water. Novapex.

[47] Aljanabi S.M., Martinez I. (1997). Universal and rapid salt-extraction of high quality genomic DNA for PCR-based techniques. Nucleic Acids Res..

[48] Bolger A.M., Lohse M., Usadel B. (2014). Trimmomatic: A flexible trimmer for Illumina sequence data. Bioinformatics.

[49] Bernt M., Donath A., Jühling F., Externbrink F., Florentz C., Fritzsch G., Pütz J., Middendorf M., Stadler P.F. (2013). MITOS: Improved de novo metazoan mitochondrial genome annotation. Mol. Phylogenet. Evol..

[50] Xia X. (2018). DAMBE7: New and improved tools for data analysis in molecular biology and evolution. Mol. Biol. Evol..

[51] Hassanin A., Leger N., Deutsch J. (2005). Evidence for multiple reversals of asymmetric mutational constraints during the evolution of the mitochondrial genome of Metazoa, and consequences for phylogenetic inferences. Syst. Biol..

[52] Kumar S., Stecher G., Li M., Knyaz C., Tamura K. (2018). MEGA X: Molecular evolutionary genetics analysis across computing platforms. Mol. Biol. Evol..

[53] Rozas J., Ferrer-Mata A., Sánchez-Delbarrio J.C., Guirao-Rico S., Librado P., Ramos-Onsins S.E., Sánchez-Gracia A. (2017). DnaSP 6: DNA sequence polymorphism analysis of large data sets. Mol. Biol. Evol..

[54] Ronquist F., Teslenko M., Van Der Mark P., Ayres D.L., Darling A., Höhna S., Larget B., Liu L., Suchard M.A., Huelsenbeck J.P. (2012). MrBayes 3.2: Efficient Bayesian phylogenetic inference and model choice across a large model space. Syst. Biol..

[55] Minh B.Q., Schmidt H.A., Chernomor O., Schrempf D., Woodhams M.D., Von Haeseler A., Lanfear R. (2020). IQ-TREE 2: New models and efficient methods for phylogenetic inference in the genomic era. Mol. Biol. Evol..

[56] Nguyen L., Schmidt H.A., Von Haeseler A., Minh B.Q. (2015). IQ-TREE: A fast and effective stochastic algorithm for estimating maximum-likelihood phylogenies. Mol. Biol. Evol..

[57] Kalyaanamoorthy S., Minh B.Q., Wong T.K., Von Haeseler A., Jermiin L.S. (2017). ModelFinder: Fast model selection for accurate phylogenetic estimates. Nat. Methods.

[58] Posada D., Crandall K.A. (1998). MODELTEST: Testing the model of DNA substitution. Bioinformatics.

[59] Swofford D.L. (2003). PAUP* Phylogenetic Analysis Using Parsimony (* and Other Methods). Version 4. http://paup.csit.fsu.edu/.

[60] White T.R., Conrad M.M., Tseng R., Balayan S., Golding R., de Frias Martins A.M., Dayrat B.A. (2011). Ten new complete mitochondrial genomes of pulmonates (Mollusca: Gastropoda) and their impact on phylogenetic relationships. BMC Evol. Biol..

[61] Xie G., Köhler F., Huang X., Wu R., Zhou C., Ouyang S., Wu X. (2019). A novel gene arrangement among the Stylommatophora by the complete mitochondrial genome of the terrestrial slug Meghimatium bilineatum (Gastropoda, Arionoidea). Mol. Phylogenet. Evol..

[62] Feng J., Guo Y., Yan C., Ye Y., Li J., Guo B., Lü Z. (2020). Comparative analysis of the complete mitochondrial genomes in two limpets from Lottiidae (Gastropoda: Patellogastropoda): Rare irregular gene rearrangement within Gastropoda. Sci. Rep..

[63] Lavrov D.V., Brown W.M., Boore J.L. (2000). A novel type of RNA editing occurs in the mitochondrial tRNAs of the centipede Lithobius forficatus. Proc. Natl. Acad. Sci. USA.

[64] Yang Y., Liu H., Qi L., Kong L., Li Q. (2020). Complete mitochondrial genomes of two toxin-accumulated *Nassariids* (Neogastropoda: Nassariidae: *Nassarius*) and their implication for phylogeny. Int. J. Mol. Sci..

[65] Zhong S., Huang L., Huang G., Liu Y., Xu W. (2019). The first complete mitochondrial genome of Melongenidae from *Hemifusus tuba* (Neogastropoda: Buccinoidea). Mitochondrial DNA Part B.

[66] Beagley C.T., Okimoto R., Wolstenholme D.R. (1998). The mitochondrial genome of the sea anemone *Metridium senile* (Cnidaria): Introns, a paucity of tRNA genes, and a near-standard genetic code. Genetics.

[67] Searle J.B. (2000). Phylogeography—The history and formation of species. Heredity.

[68] Shao R., Campbell N.J., Schmidt E.R., Barker S.C. (2001). Increased rate of gene rearrangement in the mitochondrial genomes of three orders of hemipteroid insects. Mol. Biol. Evol..

[69] Bouchet P., Rocroi J.P., Frýda J., Hausdorf B., Ponder W., Valdes A., Warén A. (2005). A nomenclator and classification of gastropod family-group names. Malacologia.

[70] Lemarcis T., Fedosov A.E., Kantor Y.I., Abdelkrim J., Zaharias P., Puillandre N. (2022). Neogastropod (Mollusca, Gastropoda) phylogeny: A step forward with mitogenomes. Zool. Scr..

[71] Colgan D.J., Ponder W.F., Beacham E., Macaranas J. (2007). Molecular phylogenetics of Caenogastropoda (gastropoda: Mollusca). Mol. Phylogenet. Evol..

[72] Oliverio M., Modica M.V. (2010). Relationships of the haematophagous marine snail Colubraria (Rachiglossa: Colubrariidae), within the neogastropod phylogenetic framework. Zool. J. Linn. Soc..

[73] Barco A., Claremont M., Reid D.G., Houart R., Bouchet P., Williams S.T., Cruaud C., Couloux A., Oliverio M. (2010). A molecular phylogenetic framework for the Muricidae, a diverse family of carnivorous gastropods. Mol. Phylogenet. Evol..

[74] Kantor Y.I., Fedosov A.E., Kosyan A.R., Puillandre N., Sorokin P.A., Kano Y., Clark R., Bouchet P. (2022). Molecular phylogeny and revised classification of the Buccinoidea (Neogastropoda). Zool. J. Linn. Soc..

[75] Galindo L.A., Puillandre N., Utge J., Lozouet P., Bouchet P. (2016). The phylogeny and systematics of the Nassariidae revisited (Gastropoda, Buccinoidea). Mol. Phylogenet. Evol..

[76] Puillandre N., Samadi S., Boisselier M., Sysoev A.V., Kantor Y.I., Cruaud C., Couloux A., Bouchet P. (2008). Starting to unravel the toxoglossan knot: Molecular phylogeny of the “turrids”(Neogastropoda: Conoidea). Mol. Phylogenet. Evol..

[77] Yang M., Dong D., Li X. (2021). The complete mitogenome of *Phymorhynchus* sp. (Neogastropoda, Conoidea, Raphitomidae) provides insights into the deep-sea adaptive evolution of Conoidea. Ecol. Evol..

[78] Parins-Fukuchi C., Stull G.W., Smith S.A. (2021). Phylogenomic conflict coincides with rapid morphological innovation. Proc. Natl. Acad. Sci. USA.

